# Efficacy of Percutaneous Treatment of Primary Aneurysmal Bone Cysts (ABCs): A Systematic Review and Meta-Analysis

**DOI:** 10.3390/jcm12237213

**Published:** 2023-11-21

**Authors:** Ramy Samargandi, Muhand Alkameshki, Mohammed Barnawi, Khalid Alzahrani, Othman Iskander, Quentin Nicolas, Bandar Hetaimish, Julien Berhouet, Louis-Romée Le Nail

**Affiliations:** 1 Service de Chirurgie Orthopédique et Traumatologique, CHRU Trousseau, Faculté de Médecine de Tours, Université de Tours, 1C Avenue de la République, 37170 Chambray-les-Tours, France or rsamargandi@uj.edu.sa (R.S.); dr.myyk@hotmail.com (M.A.); q.nicolas@chu-tours.fr (Q.N.); lr.lenail@chu-tours.fr (L.-R.L.N.); 2 Department of Orthopedic Surgery, Faculty of Medicine, University of Jeddah, Jeddah 23218, Saudi Arabia; bmhetaimish@uj.edu.sa; 3 Department of Surgery, Faculty of Medicine, Al-Baha University, Al-Aqiq 65522, Saudi Arabia; mbarnawi@bu.edu.sa (M.B.); khaalzahrani@bu.edu.sa (K.A.); 4 Department of Surgery, Faculty of Medicine, Jazan University, Jazan 45142, Saudi Arabia; oiskander@jazanu.edu.sa

**Keywords:** aneurysmal bone cysts, injection, doxycycline, Ethibloc, sclerotherapy, embolization, polidocanol, calcitonin

## Abstract

Background: Percutaneous treatment for primary aneurysmal bone cysts (ABCs) has been widely accepted. The study aimed to evaluate the efficacy of various sclerotherapy agents on patients with primary ABCs. Methods: A meta-analysis of relevant studies. A systematic search was conducted on five databases, resulting in the inclusion of 25 studies with different percutaneous agents. Results: A total of 729 patients with primary ABCs were included. Patients were administered with Ethibloc, doxycycline, embolization, alcohol, polidocanol, and calcitonin with methylprednisolone, respectively. Overall, 542 (74.3%) patients with ABCs had complete healing, 120 (16.4%) had partial healing, 44 (6%) had no-ossification or failure, and 26 (3.5%) had a recurrence. However, there was a total of 45 (6.1%) patients who had surgical curettage after sclerotherapy. Among the sclerotherapy agents, doxycycline showed highly effective results with minimal complications and recurrence, but it required multiple injections per patient. Ethibloc and embolization also proved to be highly effective with fewer injections required but had a higher rate of complications. Absolute alcohol, polidocanol, and calcitonin with methylprednisolone had similar efficacity and favorable success with fewer complications and fewer injections. Conclusion: Percutaneous treatment showed promising results in treating primary ABCs. However, more robust research is needed to establish the best approach for sclerotherapy in clinical practice and to address the limitations of the current literature.

## 1. Introduction

Aneurysmal bone cysts (ABCs) were first defined in 1942 by Jaffe and Lichtenstein as self-destructive, benign, and locally aggressive bone growing tumor, which accounts for 1% of all bone tumors [1,2]. ABCs commonly occurs in children or adolescents, and most of the lesions appear before the age of 30 years [2]. The prevalence of ABCs is 1.4 per 100,000. The symptoms observed with this lesion are pain, swelling, deformity, and pathological fracture in severe cases [3]. The diagnosis is initially made using radiographic imaging, but the final and definitive diagnosis is confirmed through histopathology [4,5].

ABC treatment modalities can be subdivided broadly into three categories such as surgical, percutaneous, and others. Surgical treatment includes intralesional curettage with or without adjuvants (high-speed burr, argon beam, phenol, en bloc resection) [6,7]. Percutaneous treatment includes embolization or sclerotherapy. Other treatment modalities include radiation therapy, radionuclide ablation, or the systemic application of bisphosphonates or denosumab [8].

While surgical treatment is generally considered the standard approach for ABCs, it can present challenges in specific locations like the axial spine. Among children, the operative complications following curettage or surgical intervention are notably high [3,9,10]. Moreover, the recurrence rate after surgical treatment ranges from 18% to 59% [6,11].

Currently, percutaneous sclerotherapy and embolization for ABCs are widely accepted alternative treatments for primary ABCs or recurrent ABCs after previous surgical treatment, as they are associated with decreased morbidity and recurrence rates. Studies have reported a recurrence of 5–6% within a period of 18–24 months [3,9]. Studies have suggested that percutaneous treatment can be used as a first line of treatment or as an accepted alternative to surgery as it has been demonstrated to have many advantages over conventional surgery, including lower complications and morbidity and better functional outcomes, with a comparable healing rate [3,9,11].

Embolization is a minimally invasive procedure that blocks the arterial supply to a tumor using variable agents. It can be used as an adjuvant to decrease hemorrhage, or as an alternative treatment. It is usually performed under local anesthesia, but general anesthesia may be used. There have been few postoperative complications reported, and the most important ones are bleeding, infection, and nerve damage [2,12].

In the current literature, there are many agents have been described as sclerotherapy agents including polidocanol, doxycycline, calcitonin with methylprednisolone, absolute alcohol, and Ethibloc [13,14,15,16].

Despite the wide range of agents utilized in treatments, there is a shortage of literature comparing percutaneous treatment options to determine the most effective approach. Therefore, this systematic review and meta-analysis systematically provides evidence to determine the effectiveness of different sclerotherapy agents and embolization in treating primary ABCs. This study also presents evidence on recurrence rates, complication rates, types of complications, and the number of injections required for the treatment of ABCs with each sclerotherapy intervention.

## 2. Materials and Methods

### 2.1. Searching

A comprehensive systematic search was conducted in 5 databases, PubMed, Cochrane Central Register of Controlled Trials (CENTRAL), Clinicaltrials.eu, Clinicaltrials.gov, and ICTRP, using the search terms ‘aneurysmal bone cyst’, ‘sclerotherapy’, ‘embolization’, ‘Ethibloc’, ‘polidocanol’, ‘doxycycline’, ‘alcohol’, ‘calcitonin’, and ‘calcitonin with methylprednisolone’. The search terms were combined using Boolean operators. Back reference searching was also conducted from previous systematic review papers. The search was restricted to the English language and citations published after the year 2000.

### 2.2. Selection and Screening

A two-stage screening was conducted. First, the title and abstract were screened, followed by full-text screening using predesigned inclusion and exclusion criteria. The inclusion criteria for the study were based on the PICO format.

**Inclusion criteria**:

- Population: Studies that had primary percutaneous ABC treatments in which interventions were evaluated with a mean follow-up period of at least 18 months.
- Intervention: Studies that used embolization, doxycycline/albumin, calcitonin with methylprednisolone, alcohol, Ethibloc, or polidocanol for treating primary ABCs.
- Comparator: Studies comparing any sclerotherapy agents, either using surgical or no intervention.
- Outcome: Complete healing, partial healing, failure, recurrence, number of injections administered for healing, and complications.
- Studies: Retrospective or prospective studies, RCTs, clinical trials. In studies with multiple intervention groups, only patients administered any sclerotherapy agents or embolization would be selected.
- Language: English.
- Time: Published from 2000 to 2021.

**Exclusion criteria:**

- Population: Studies that had primary percutaneous ABC treatments and evaluations of interventions with a mean follow-up period less than 18 months. Studies assessing secondary ABCs.
- Intervention: Studies that used any sclerotherapy as secondary treatment for primary ABCs, surgical therapy, non-injection therapy, or only mixed treatments.
- Comparator: Studies comparing other modalities apart from our interventions of interest.
- Outcome: Other outcomes apart from our interventions of interest.
- Studies: Case reports, letters, narrative reviews, or systematic reviews.
- Language: Non-English.
- Time: Studies published before 2000.

### 2.3. Data Extraction and Quality Assessment

Complete data extraction and quality assessment based on the study types were conducted. Data were extracted regarding the country published in, study setting, population type, sample size, treatment, ABC site, follow-up time, age, number of injections, complete ossification or healing, partial ossification, recurrence, failure, post-therapy surgery, and complications. In case studies with multiple interventions, data have been reported and extracted separately. For the quality assessment, the NIH study quality assessment tool was used for case series studies, before–after studies with no control group, and controlled trials studies. Each type of tool used with the studies has its domain of tool in which overall studies are marked as low-, fair-, or good-quality studies [17]. A quality score was provided for the selected studies on a scale of 0 to 14, with 0 representing a study that failed to meet any criteria and 14 signifying a study that did meet all the criteria. The total number of points revealed a study’s overall quality. The quality of the studies was rated as being low (scoring < 4), fair (5–8), or good (>8).

### 2.4. Narrative Synthesis and Meta-Analysis

Following data extraction and quality assessment, narrative synthesis was conducted. An overall narrative synthesis was performed based on the studies. The synthesis was performed based on the various types of sclerotherapies and embolization used compared with complications, overall complete healing, and number of injections used for complete or partial healing. A meta-analysis was also performed using the random-effect model, and the proportion of the effect (event rate) and the 95% confidence interval (95% CI) were calculated. Heterogeneity among the studies was checked using the degree of heterogeneity (I2 statistics) and a forest plot. An I2 more than 75% is considered high heterogeneity, and less than 25% is considered low heterogeneity. A subgroup analysis of different sclerotherapy and embolization therapies was conducted. All the meta-analyses were performed using the Openmetaanalyst software (OpenMeta[Analyst], version 3.X).

## 3. Results

**Figure 1:**
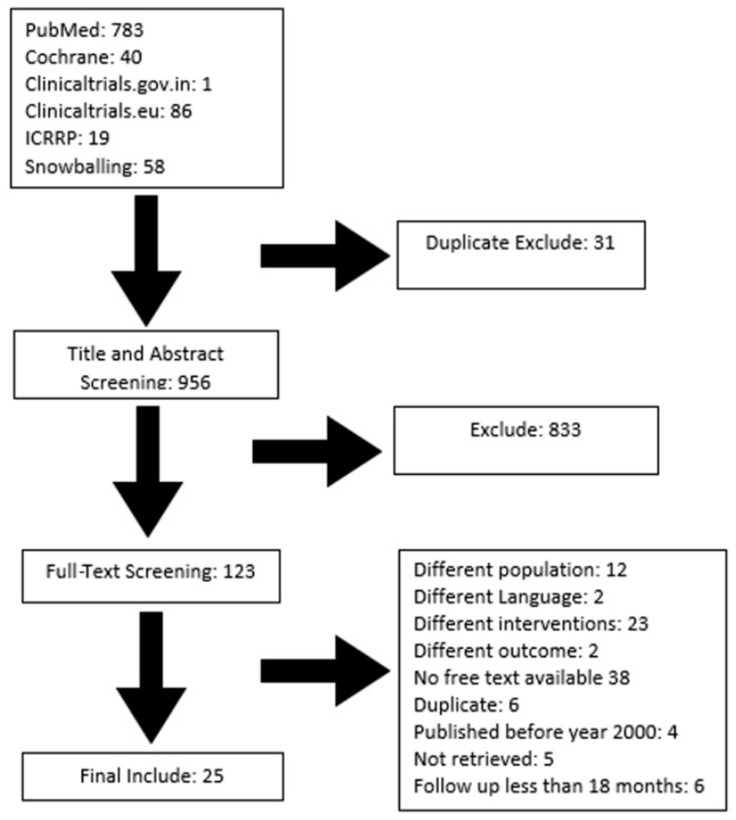
PRISMA diagram for selection of studies.

The study was conducted according to the PRISMA guidelines. Following the search of PUBMED, Cochrane, and clinical trial sites, 987 citations were retrieved, out of which 25 studies were included using our pre-designed inclusion and exclusion criteria, as shown in Figure 1. Overall, 729 patients with primary ABCs were included for analysis. A total of 118 patients were administered injection (Inj.) Ethibloc, 57 patients were administered Inj. doxycycline, 110 patients were treated with embolization, 83 patients were administered Inj. alcohol, 314 patients were administered Inj. polidocanol, and 47 patients were administered a combination of calcitonin with methylprednisolone as shown in Table 1.

### 3.1. Study Characteristics

The majority of the studies were retrospective, with only one randomized control trial and one prospective study included. The characteristics of the studies are detailed in Table 1. In terms of age, the majority of the studies included patients aged below 18 years old, with a mean age of 12.9 years (±3.7). The location of the lesion varied among the patients and included the humerus, pelvis, femur, spine, and other locations. The mean least follow-up observed was 25.5 months, and the longest follow-up observed was 84 months, with details of patient characteristics outlined in Table 2. The study quality was assessed using the NIH quality assessment tool for case studies as shown in Table 1. The quality of all included studies was assessed independently by two authors, and it was observed that 7 studies were of poor quality, 11 studies were of fair quality, and 7 studies were of good quality.

### 3.2. Study Outcomes

Study outcome data were extracted based on complete ossification or more than 75% healing, partial ossification, no-ossification or failure, recurrence, post-therapy surgical treatment, complications, and number of injections required to be administered.

Overall, 74.3% patients (n = 542) with ABCs had complete healing or more than 75% reduction in cyst volume, 16.4% patients (n = 120) with ABCs had partial healing (25 to 75% reduction in cyst volume), 6% patients (n = 44) with ABCs had no-ossification or failure, and 3.56% patients (n = 26) with ABCs had a recurrence. However, there was a total of 6.1% patients (n = 45) who had surgical curettage after sclerotherapy (Table 3).

### 3.3. Narrative Synthesis

The highest complete healing rate was observed in the doxycycline/albumin group, at 95% (n = 54), while the lowest was in the polidocanol group, at 70.4% (n = 221). Among the embolization group, the healing rate was 79% (n = 87). We found that the highest complication rate was among those who received an Ethibloc injection, at 52.5% (n = 62). However, in the calcitonin with methylprednisolone group, no patients presented with any complications.

Overall, the main complications observed in all the intervention groups were fever, indurations, skin rash, skin necrosis, edema, cutaneous fistula, and pain, as shown in Table 4. The recurrence rate varied among the various intervention groups, with 14.5% (n = 16) in the embolization group and no recurrence observed in the alcohol and calcitonin with methylprednisolone groups. Outcomes are detailed in Table 4.

There was a lot of heterogeneity among the number of injections administered among various interventions or sclerotherapy groups as shown in Table 5. The mean number of injections required across all the sclerotherapy groups for complete or partial healing was 1.35 [1 to 4] in the Ethibloc group, 3.95 [1 to 14] in the doxycycline/albumin group, 1.6 [1 to 7] in the embolization group, 1.7 [1 to 4] in the alcohol group, 2.8 [1 to 12] in the polidocanol group, and 2.8 [1 to 7] in the calcitonin with methylprednisolone group.

### 3.4. Meta-Analysis

**Figure 2:**
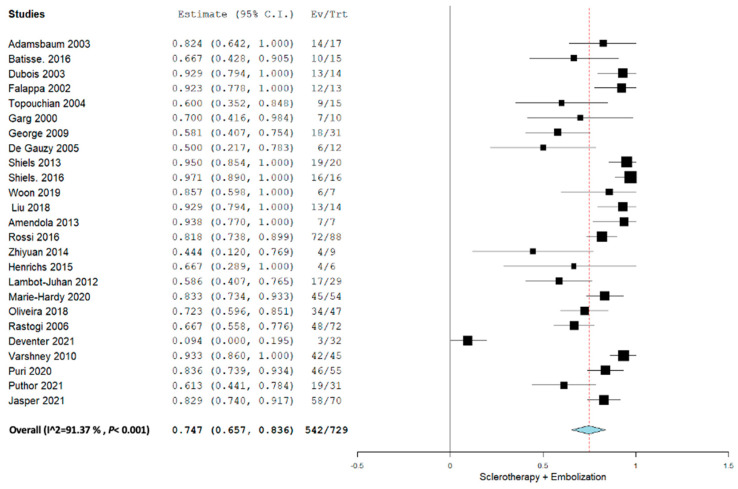
Overall pooled efficacy of all sclerotherapies + embolization.

Applying the random effect model, the overall pooled proportion size for complete healing for all the sclerotherapy and embolization agents was 0.747 (95% CI, 0.657–0.836) with a heterogeneity of 91.37% as shown in Figure 2. The overall pooled proportion for only sclerotherapy agents was 0.746 (95% CI, 0.643–0.848) with a heterogeneity of 92.59%.

**Figure 3:**
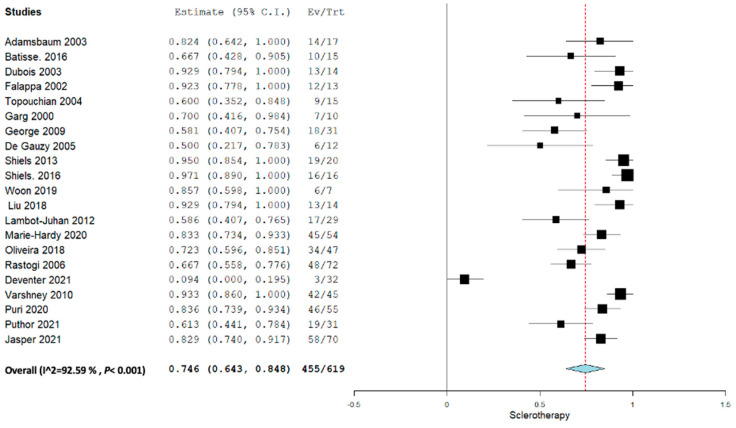
Overall pooled efficacy of all sclerotherapies.

**Figure 4:**
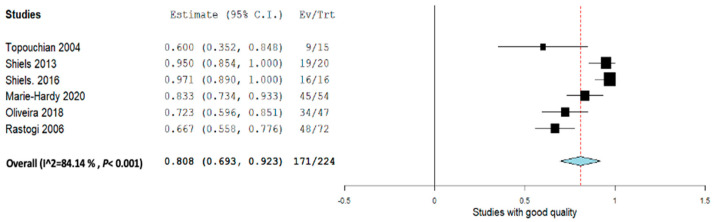
Overall pooled efficacy among good-quality studies.

Excluding the poor- and fair-quality studies while taking into consideration the good-quality studies, the overall pooled proportion size for complete healing for all the sclerotherapies was 0.808 (95% CI,0.693–0.923) with a heterogeneity of 84.14% as shown in Figure 3. Overall, all sclerotherapy showed significant effectiveness but with a high amount of heterogeneity even after sensitivity analysis, and thus the results should be used with caution (Figure 4).

**Figure 5:**
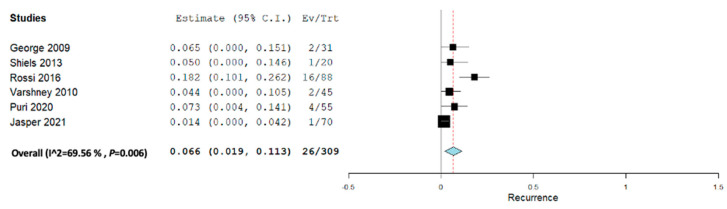
Overall pooled recurrence all sclerotherapies + embolization.

The recurrence rate as shown in Figure 5 showed significantly low recurrence with a medium amount of heterogeneity among the studies. The pooled recurrence rate was 0.66 (95% CI, 0.019–0.113) with a heterogeneity of 69.56%.

### 3.5. Subgroup Analysis

**Figure 6:**
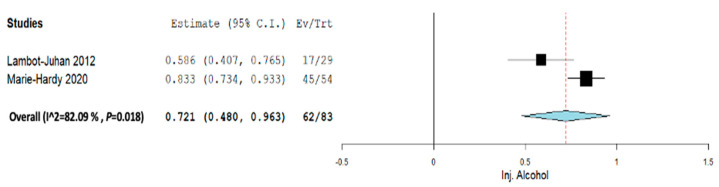
Overall pooled efficacy for Inj. alcohol.

**Figure 7:**
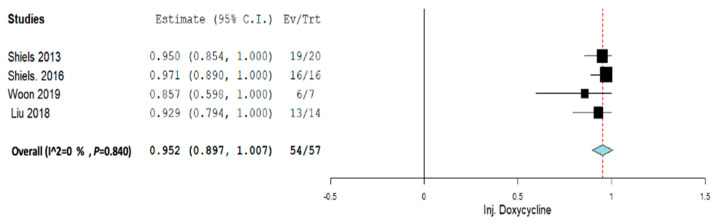
Overall pooled efficacy for Inj. doxycycline.

**Figure 8:**
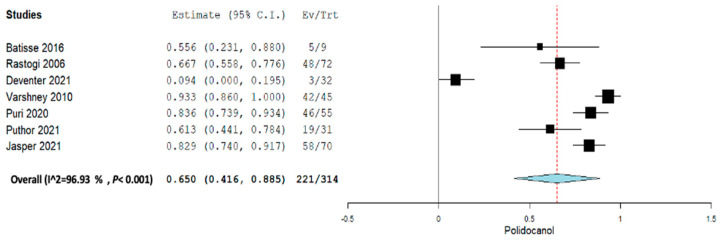
Overall pooled efficacy for Inj. polidocanol.

**Figure 9:**
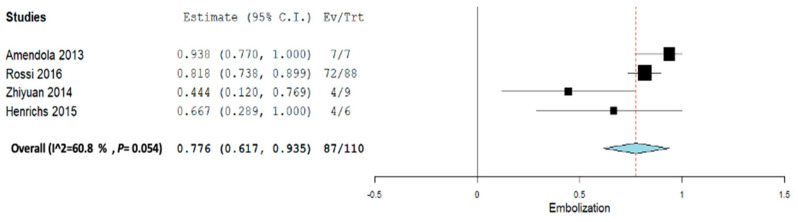
Overall pooled efficacy for embolization therapy.

**Figure 10:**
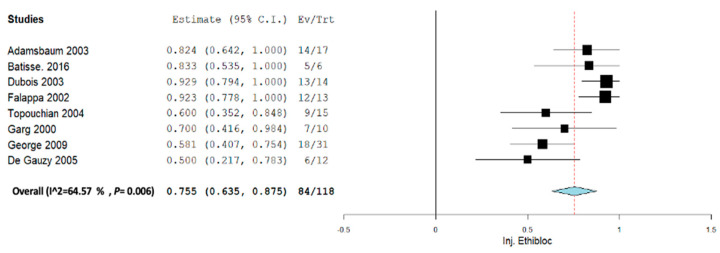
Overall pooled efficacy for Inj. Ethibloc.

Subgroup analysis was performed across each intervention for Inj. alcohol, Inj. doxycycline, Inj. Polidocanol, and embolization. Across all the subgroup analyses, a random-effect model was used. In the Inj. alcohol group, the pooled proportion size for complete healing was 0.721 (95% CI, 0.480–0.963) with a heterogeneity of 82.09% as shown in Figure 6. In the Inj. doxycycline group, the pooled proportion size for complete healing was 0.952 (95% CI, 0.897–1.007) with a heterogeneity of 0% as shown in Figure 7. In the Inj. polidocanol group, the pooled proportion size for complete healing was 0.650 (95% CI, 0.416–0.885) with a heterogeneity of 96.93% as shown in Figure 8. In the embolization group, the pooled proportion size for complete healing was 0.776 (95% CI, 0.617–0.935) with a heterogeneity of 60.8% as shown in Figure 9. In the Inj. Ethibloc group, the pooled proportion size for complete healing was 0.755 (95% CI, 0.635–0.875) with a heterogeneity of 64.57% as shown in Figure 10.

Overall, all five sclerotherapy interventions showed significant effectiveness in terms of complete healing. However, due to the high amount of heterogeneity in the polidocanol and alcohol groups and the moderate amount of heterogeneity in the embolization group and Ethibloc group, the effectiveness must be taken into consideration with caution. The Inj. doxycycline group showed highly effective results with the least amount of heterogeneity.

During the analysis of subgroups, the group involving calcitonin with methylprednisolone injection was not considered as a subgroup. This decision was made because there was only one study included in this meta-analysis, and it had a limited number of patients. However, after conducting a search, we found two more studies. Unfortunately, these additional studies did not meet our inclusion criteria.

## 4. Discussion

Percutaneous treatment of aneurysmal bone cysts (ABCs) is considered a favorable alternative to conventional therapy, yielding comparable outcomes but with a less challenging operative technique and, consequently, fewer complications. Nevertheless, numerous agents are employed as sclerosing agents, each exhibiting varying degrees of efficacy [38].

This meta-analysis aims to assess the clinical effectiveness of sclerotherapy for primary ABC patients. While previous systematic reviews and meta-analyses have been conducted [11,38,39,40], this updated meta-analysis was necessitated by concerns regarding publication bias. Additionally, this review specifically examines complications associated with different types of sclerotherapies and the number of injections required for ABC healing.

Overall, sclerotherapy has proven to be effective in reducing the size of cysts as well as healing tumors. The previous systematic review showed efficacious results in healing tumors [3,11]. The meta-analysis showed significant results overall regarding the healing rates of ABCs. Upon conducting the sensitivity analysis, sclerotherapy still possessed significant results. The overall pooled proportion of complete healing for all sclerotherapy and embolization treatments was 0.747 (95% CI, 0.657–0.836), which means that about 75% of patients healed completely. However, we found that there was high heterogeneity between the studies, meaning that there was a lot of variation in the results of the studies. This heterogeneity could be due to differences in the patient populations, treatments, or methods used in the studies.

In our study, the overall recurrence rate after an 18-month follow-up period was 3.4%. It is worth noting that the surgical technique, which is considered the gold standard treatment for ABCs, has a recurrence rate ranging from 18% to 59%, primarily attributed to incomplete cyst removal [10]. In contrast, across all the sclerotherapy methods, the overall complication rate was 20%. Sclerotherapy offers several advantages, including lower morbidity, accessibility in challenging surgical access areas, reduced technique sensitivity, and greater compatibility with patients [3,10,11]

In the doxycycline/albumin injection group, impressive efficacy rates were observed, with a 94.7% rate of complete healing and a mere 1.7% recurrence rate. However, it is noteworthy that the complication rate stood at 3.5%, with the primary complication being focal skin necrosis. Despite its effectiveness and low recurrence rate, there is one drawback regarding its utilization; it necessitates a mean of 3.5 injections, with a maximum of 14 injections required, which is considered a significant limitation.

Both polidocanol and alcohol agents have shown promising results, with efficacies of 70.6% and 74.6%, respectively, and low recurrence rates. These agents are considered safe due to their mild complication profiles compared to others. Furthermore, when compared to doxycycline, both require a lower number of injections, with a mean of 2.8 injections for polidocanol and 1.7 for alcohol. However, it is worth noting that the efficacy of polidocanol could potentially be improved by excluding the article by Deventer et al., as it appears to be an outlier in this meta-analysis. In most other articles, the efficacy rates are notably higher, such as those by Jasper et al. (83%), Rastogi et al. (97%), Varshney et al. (98%), Puri et al. (82%), and Puthoor et al. (100%). Embolization has shown good results with 79% efficacity, but there was a 14.5% recurrence rate, and the complication rate was 4.5% with observation of major complications such as paralysis. It essential to consider that the bulk of the patients in these studies had axial spine ABCs.

This study also found out that the highest rate of complications was about 52% for the Ethibloc group while the rest of the sclerotherapy agents and the embolization group had complication rates less than 20%. Most of the complications across the various therapies included fever, pain, induration, swelling, and serious local complications such as skin necrosis and cutaneous fistula. Most of the local adverse events seen were due to uneven distribution of the sclerotherapy agents, which leads to incomplete cure and can be minimized using proper biopsy and radiographic techniques [10]. Ethibloc has been withdrawn from the market and is no longer approved by the FDA due to its severe side effects, such as pulmonary embolism [22,38]. The least or negligible recurrences were observed for the alcohol, doxycycline/albumin, and calcitonin with methylprednisolone Inj. groups and were very low compared to recurrences for surgical techniques. The mean number of injections required for healing or complete ossification is 2.15 among the sclerotherapy agents and 1.6 for embolization therapy. The highest number of injections were required among the doxycycline/albumin group, ranging from 1 to 14 injections with an average of 3.95 injections. Due to the high numbers of injections, this sclerotherapy agent is not accepted as the only mode of treatment. However, it has been suggested to use sclerotherapy in most inaccessible surgical areas [10].

This study also had strengths and weaknesses in terms of study methodology. First, a robust search was conducted in all five databases followed by strict screening using the inclusion and exclusion criteria. Moreover, the study mean follow-up timing was set to at least 18 months, as most recurrences of ABCs occur between 18 and 24 months [10,18]. This criterion would lead us to find evidence regarding the recurrence of ABCs following sclerotherapy. Another key strength of the study was that quality checking of all the studies was conducted and taken into consideration while assessing their efficacy. On the other hand, all steps of this review process were conducted by a single reviewer, and no authors were contacted during the review process. However, to minimize bias, each and every step was double-checked. Secondly, most of the studies included in this review were case studies and retrospective studies. According to the hierarchy of evidence, RCTs and clinical trials provide very good clinical evidence. Lastly, the presence of substantial heterogeneity among the included studies is a significant limitation in our analysis. We recognize the importance of exercising caution when interpreting our results due to this observed heterogeneity. Heterogeneity can introduce variability into the findings, potentially influencing the overall reliability of our conclusions. To address this issue, we conducted a subgroup analysis aimed at exploring potential sources of heterogeneity. However, it is important to note that despite these efforts, some level of heterogeneity may persist. This is largely due to the inherent diversity among the included studies in terms of study design, population, and other factors that influence outcomes.

## 5. Conclusions

Overall, excluding the heterogeneity among studies and the study types, sclerotherapy was highly effective in controlling or healing ABCs with minimal complications and a low recurrence rate. In particular, doxycycline/albumin provided very good evidence for controlling ABCs, and it can be used in clinical practices with minimal or negligible complications, failures, and recurrence rates, but it required at least two injections for complete healing. Embolization, on the other hand, showed high healing rates with minimal complications and recurrence and no failure rates, but it was less effective compared to sclerotherapy. However, other sclerotherapy interventions should be used with caution in clinical practices, as strong evidence is lacking to support the efficacy of these interventions. Thus, good quality RCTs or clinical trials are required to determine the clinical effectiveness of sclerotherapy and its application in the clinical setting as a definitive treatment for ABCs.

## Figures and Tables

**Table 1:** Study characteristics.

Study Name	Country	Study Design	Sample Size	Treatments/Interventions	Quality of Study
Adamsbaum 2003 [18]	France	Retrospective study	17	Inj. Ethibloc	Fair
Batisse 2016* [19]	France	Retrospective study	6	Inj. Ethibloc	Poor *
Dubois 2003 [20]	Canada	Retrospective study	14	Inj. Ethibloc	Fair
Falappa 2002 [21]	Italy	Retrospective study	13	Inj. Ethibloc	Fair
Topouchian 2004 [22]	France	Retrospective study	15	Inj. Ethibloc	Good
Garg 2000 [23]	UK	Retrospective study	10	Inj. Ethibloc	Poor
George 2009 [24]	UK	Retrospective study	31	Inj. Ethibloc	Good
De Gauzy 2005 [25]	France	Retrospective study	12	Inj. Ethibloc	Poor
Shiels 2013 [15]	USA	Retrospective study	20	Inj. doxycycline/albumin	Fair
Shiels 2016 [26]	USA	Retrospective study	16	Inj. doxycycline/albumin	Fair
Woon 2019 [27]	New Zealand	Retrospective study	7	Inj. doxycycline/albumin	Poor
Liu 2019 [28]	China	Retrospective study	14	Inj. doxycycline/albumin	Fair
Rossi 2016 [29]	Italy	Retrospective study	88	Embolization	Fair
Cheng 2014 [30]	China	Retrospective study	9	Embolization	Poor
Henrichs 2015 [31]	Germany	Retrospective study	6	Embolization	Poor
Amendola 2013 [2]	Italy	Retrospective study	7	Embolization	Poor
Lambot-Juhan 2012 [32]	France	Retrospective study	29	Inj. alcohol	Good
Marie-Hardy 2020 [33]	France	Retrospective study	54	Inj. alcohol	Fair
Oliveira 2018 [34]	Brazil	Retrospective study	47	Inj. calcitonin with methylprednisolone	Fair
Batisse 2016 * [19]	France	Retrospective study	9	Inj. polidocanol	Poor *
Rastogi 2006 [35]	India	Prospective study	72	Inj. polidocanol	Fair
Deventer 2021 [1]	Germany	Retrospective study	32	Inj. polidocanol	Good
Varshney 2010 [9]	India	RCT	45	Inj. polidocanol	Good
Puri 2020 [36]	India	Retrospective study	55	Inj. polidocanol	Fair
Puthoor 2021 [10]	India	Retrospective study	31	Inj. polidocanol	Good
Jasper 2021 [37]	Netherlands	Retrospective study	70	Inj. polidocanol	Good

* = same study but different agents.

**Table 2:** Patient characteristics.

Study Name	Mean Age (Year)	ABC Site	Mean Follow-Up Time (Months)
Adamsbaum 2003 [18]	8 [2–18]	Pelvis, femur, humerus, fibula, clavicle, ulna, metacarpal	60 [18–132]
Batisse 2016 [19]	12 [3–17]	Femur, humerus, foot and ankle, pelvis, spine, fibula	35 [3–96]
Dubois 2003 [20]	10.71 [2.5–15]	Metatarsal bone, humerus, paranasal sinus, mandible, humerus, acetabulum, fibula, ribs, sacrum	57.3 [24–108]
Falappa 2002 [21]	12 [5–23]	Femur, tibia, humerus, pelvis, ankle, foot	25.5 [6–67]
Topouchian 2004 [22]	10.3 [3–15]	Femur, tibia, humerus, pelvis	80 [47–116]
Garg 2000 [23]	11.8 [4–16]	Ilium, tibia, humerus, femur	29.8 [6–60]
George 2009 [24]	[3–16]	Humerus, femur, tibia, fibula, pelvis	54 [22–90]
De Gauzy 2005 [25]	9.6 [5–13.5]	Tibia, femur, fibula, femur, metatarsal bone, clavicle, humerus	64 [24–81]
Shiels 2013 [15]	10 [3–18]	Humerus, spine, clavicle, fibula, femur, ulna, tibia, scapula	38 [24–63]
Shiels 2016 [26]	7.1 [2–15]	Tibia, humerus, fibula, femur, ulna	42 [24–67]
Woon 2019 [27]	12.42 [8–18]	Spine, sacrum, pelvis, femur	26 [14–60]
Liu 2019 [28]	18.4 [6–36]	Spine	30.7 [24–50]
Rossi 2016 [29]	16 [3–60]	Pelvis, sacrum, spine, femur, tibia, fibula, humerus, scapula, clavicle	84 [30–156]
Cheng 2014 [30]	24 [11–43]	Sacrum	32 [24–47]
Henrichs 2015 [31]	13.7 [8–18]	Sacrum	36.5 [14–56]
Amendola 2013 [2]	17 [6–41]	Spine	46 [24–64]
Lambot-Juhan 2012 [32]	[2–16]	Ulna, pelvis, femur, humerus, tibia, phalanx, spine, radius, clavicle, scapula	30 [3–90]
Marie-Hardy 2020 [33]	9.6	Pelvis, calcaneum, clavicle, cuboid, femur, fibula, humerus, metacarpal bone, cervical spine, radius, tibia	51 [24–117]
Oliveira 2018 [34]	17.5 [4–54]	Scapula, clavicle, humerus, hand, femur, tibia, foot, fibula, pelvis	45.5 [24–59.5]
Rastogi 2006 [35]	15.6 [3–38]	Upper limb, lower limb, axial skeleton	34 [25.5–80]
Deventer 2021 [1]	16.88	Ankle, foot, clavicle, scapula, pelvis	40.5 [13.1–104]
Varshney 2010 [9]	21.3	Humerus, forearm, femur, tibia, fibula, pelvis, clavicle, hand, spine	52.8 [38.4–73.2]
Puri 2020 [36]	20 [1–54]	Clavicle, humerus, femur, tibia	62 [20–111]
Puthoor 2021 [10]	17.67 [4–45]	Clavicle, femur, fibula, humerus, pelvis, metacarpal bones, metatarsal bones, phalanx, radius, sacrum, scapula, talus, tibia	24
Jasper 2021 [37]	11 [3–17]	Humerus, tibia, pelvis, femur, fibula, scapula, foot, hand, clavicle, scapula, ulna	40 [18–144]

**Table 3:** Study outcomes.

Study Name	Sample Size Receiving the Treatment	Mean No. of Injections	CO * or >75% Reduction in Cyst Size	PO ** or 25–75% Reduction in Cyst Size	No-Ossification or Failure	Recurrence	Post-Therapy Surgery
Adamsbaum et al., 2003 [18]	17	1 [1–3]	14	1	2		
Batisse et al., 2016 [19] (Ethibloc group)	6	1.3 [1–2]	5	1			
Batisse et al., 2016 [19] (polidocanol group)	9	1.2 [1–2]	5	4			
Dubois et al., 2003 [20]	14	1.8 [1–4]	13	1			
Falappa et al., 2002 [21]	13	2.4 [1–4]	12	1			
Topouchian et al., 2004 [22]	15	1.1 [1–3]	9	2	4		
Garg et al., 2000 [23]	10	1.3 [1–2]	7	3			
George et al., 2009 [24]	31	1.3 [1–2]	18	11		2	
De Gauzy et al., 2005 [25]	12	1.1 [1–2]	6	3	3		3
Shiels et al., 2013 [15]	20	5.9 [1–14]	19			1	
Shiels et al., 2016 [26]	16	5.9 [2–14]	16				
Woon et al., 2019 [27]	7	1.3 [1–2]	6				1
Liu et al., 2019 [28]	14	3 [2–4]	13	1			
Amendola et al., 2013 [2]	7	2.1 [1–5]	7				
Rossi et al., 2016 [29]	88	1.3 [1–3]	72			16	
Cheng et al., 2014 [30]	9	4.1 [3–7]	4	5			
Henrichs et al., 2015 [31]	6	1.5 [1–3]	4	2			
Lambot-Juhan et al., 2012 [32]	29	1.7 [1–4]	17	9	3		4
Marie-Hardy et al., 2020 [33]	54	1.7 [1–4]	45	9			5
Oliveira et al., 2018 [34]	47	2.8 [1–7]	34	12	1		
Rastogi et al., 2006 [35]	72	3 [1–5]	48	24			
Deventer et al., 2021 [1]	32	5.7 [1–12]	3	19	10		10
Varshney et al., 2010 [9]	45	2.3 [1–5]	42			2	1
Puri et al., 2020 [36]	55	2 [1–5]	46		9	4	9
Puthoor et al., 2021 [10]	31	1.1 [1–2]	19	12			
Jasper et al., 2021 [37]	70	1.5 [1–5]	58		12	1	12

* = Complete ossification, ** = Partial ossification.

**Table 4:** Outcomes across the intervention groups.

Interventions	Sample Size	CH */≥75% Reduction in Cyst Size (%(n))	Failure/No-Ossification (%(n))	Complication Rate (%(n))	Types of Complications	Recurrence Rate (%(n))
Ethibloc Inj.	118	71% (84)	7.6% (9)	52.5% (62)	Fever + local inflammation: 16 Cutaneous fistula: 7 Intercostal arterial reflux: 1 Post-operative pain: 1 Local inflammation: 4 Small blister leakage: 1 Fever + pain: 6 Fever + pain + leakage: 2 Pulmonary embolization: 1 Fever: 10 Sterile abscess: 1 Leakage of contrast: 3 Skin rashes and edema: 6 Sterile abscess: 1 Sustained fracture following trauma: 2	1.7% (2)
Doxycycline/albumin Inj.	57	94.7% (54)	0	3.5% (2)	Focal skin necrosis: 2	1.7% (1)
Embolization	110	79% (87)	0	4.5% (5)	Skin necrosis: 2 Permanent paresthesia: 1 Pseudoaneurysm of femoral artery: 1 Hip pain and paralysis: 1	14.5% (16)
Alcohol Inj.	83	74.6% (62)	5% (4)	2.4% (2)	Nerve palsy: 1 Bradycardia during Inj.: 1	0
Polidocanol Inj.	314	70.6% (221)	10% (31)	25% (78)	Inflammatory nodule: 1 Cutaneous erythema: 1 Induration at Inj. site: 18 Hypopigmentation: 13 Local inflammatory reaction: 1 Dizziness: 1 Local induration: 37 Dizziness: 1 Local complications: 2 Other: 2	2.1% (7)
Calcitonin with methylprednisolone Inj.	47	72.3% (34)	2.1% (1)	0	None	0

* = Complete healing.

**Table 5:** Number of injections across the intervention groups.

Interventions	Sample Size	Mean No. of Inj.	Single Inj. (%(n))	Two Inj. (%(n))	Three Inj. (%(n))	Four Inj. (%(n))	Five Inj. (%(n))	Six or More Than Six Inj. (%(n))
Ethibloc Inj.	118	1.35	66% (77)	23% (27)	2% (8)	3% (4)	0	0
Doxycycline/albumin Inj.	57	3.95	5% (3)	20% (11)	20% (11)	16% (9)	14% (8)	25% (14)
Embolization	110	1.6	72% (79)	16% (18)	9% (10)	5% (5)	2% (2)	1% (1)
Alcohol Inj.	83	1.7	46% (38)	37% (31)	14% (12)	2.4% (2)	0	0
Polidocanol Inj.	314	2.8	Unclear	Unclear	Unclear	Unclear	Unclear	Unclear
Calcitonin with methylprednisolone Inj.	47	2.8	19% (9)	30% (14)	21% (10)	15% (7)	10% (5)	4% (2)

## Data Availability

The data presented in this study are available upon request from the corresponding author.
